# Oral steroids for reducing kidney scarring in young children with febrile urinary tract infections: the contribution of Bayesian analysis to a randomized trial not reaching its intended sample size

**DOI:** 10.1007/s00467-021-05117-5

**Published:** 2021-05-25

**Authors:** Liviana Da Dalt, Silvia Bressan, Floriana Scozzola, Enrico Vidal, Monia Gennari, Claudio La Scola, Mauro Anselmi, Elisabetta Miorin, Pietro Zucchetta, Danila Azzolina, Dario Gregori, Giovanni Montini

**Affiliations:** 1 Department of Women’s and Children’s Health, University of Padova, Via Giustiniani 4, 35128 Padova, Italy; 2 Pediatric Unit, Treviso Hospital, Treviso, Italy; 3 Division of Pediatrics, Department of Medicine (DAME), University Hospital of Udine, Udine, Italy; 4 Pediatric Emergency Unit, Department of Medical and Surgical Sciences (DIMEC), S. Orsola Hospital, Bologna, Italy; 5 Nephrology and Dialysis Unit, Department of Woman, Child and Urological Diseases, Azienda Ospedaliero-Universitaria Sant‘Orsola-Malpighi, Bologna, Italy; 6 Pediatric Unit, Dolo-Mirano Hospital, Dolo, Italy; 7 Nuclear Medicine Unit, Department of Medicine DIMED, University-Hospital of Padova, Padova, Italy; 8 Unit of Biostatistics, Epidemiology and Public Health, Department of Cardiac, Thoracic, Vascular Sciences and Public Health, University of Padova, Padova, Italy; 9 Pediatric Nephrology, Dialysis and Transplant Unit, Fondazione IRCCS Ca Granda, Ospedale Maggiore Policlinico, Milano, Italy; 10 Giuliana and Bernardo Caprotti Chair of Pediatrics, Department of Clinical Sciences and Community Health, University of Milano, Milano, Italy

**Keywords:** Urinary tract infections, Pyelonephritis, Children, Kidney scars, Dexamethasone

## Abstract

**Background:**

This study aimed to evaluate the effect of oral dexamethasone in reducing kidney scars in infants with a first febrile urinary tract infection (UTI).

**Methods:**

Children aged between 2 and 24 months with their first presumed UTI, at high risk for kidney scarring based on procalcitonin levels (≥1 ng/mL), were randomly assigned to receive dexamethasone in addition to routine care or routine care only. Kidney scars were identified by kidney scan at 6 months after initial UTI. Projections of enrollment and follow-up completion showed that the intended sample size could not be reached before funding and time to complete the study ran out. An amendment to the protocol was approved to conduct a Bayesian analysis.

**Results:**

We randomized 48 children, of whom 42 had a UTI and 18 had outcome kidney scans (instead of 128 planned). Kidney scars were found in 0/7 and 2/11 patients in the treatment and control groups respectively. The probability that dexamethasone could prevent kidney scarring was 99% in the setting of an informative prior probability distribution (which fully incorporated in the final inference the information on treatment effect provided by previous studies) and 98% in the low-informative scenario (which discounted the prior literature information by 50%). The probabilities that dexamethasone could reduce kidney scar formation by up to 20% were 61% and 53% in the informative and low-informative scenario, respectively.

**Conclusions:**

Dexamethasone is highly likely to reduce kidney scarring, with a more than 50% probability to reduce kidney scars by up to 20%.

**Trial registration number:**

EudraCT number: 2013-000388-10; registered in 2013 (prospectively registered)

**Graphical Abstract:**

**Figure:**
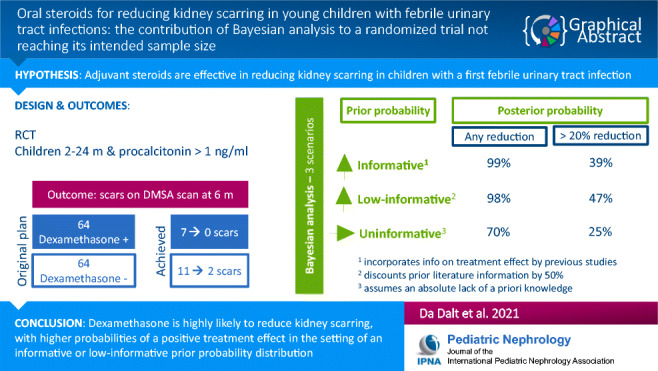
A higher resolution version of the Graphical abstract is available as Supplementary information

**Supplementary Information:**

The online version contains supplementary material available at 10.1007/s00467-021-05117-5.

## Introduction

Kidney scarring can occur in 10 to 40% of children with a urinary tract infection (UTI) despite appropriate antibiotic treatment [1, 2]. Infrequently, kidney scarring bears the potential for long-term sequelae such as hypertension, preeclampsia, and chronic kidney damage, especially in children with an underlying kidney disease [3–5].

While risk factors are still largely unknown, the inflammatory process, rather than the bacterial component, seems responsible for the permanent tissue damage of the kidney [6, 7]. On this basis, anti-inflammatory agents, such as steroids, were studied in animal models showing a reduction in scar development [8, 9]. One study of children with acute pyelonephritis showed that dexamethasone significantly decreased urinary levels of interleukin-6 and interleukin-8, suggesting a possible role in the prevention of scar formation [10].

In 2011, a randomized controlled trial (RCT) including children with pyelonephritis confirmed on acute ^99m^-Tc-dimercaptosuccinic acid (DMSA) scan found that adjuvant oral methylprednisolone was associated with a lower kidney scarring rate compared with the control group (33.3% versus 60%). [11] This study, however, included a small sample of 84 children within a wide age range (1 week to 16 years of age) and had an unbalanced ratio between the study arms (19 patients in the steroid group and 64 in the placebo group). In addition, the prevalence of kidney scarring was much higher than previously reported, as it included children with extensive pyelonephritis on acute DMSA scan, which is no longer recommended in the management of pediatric first febrile UTIs. Quite recently, Shaikh et al. randomized 546 children with suspected UTI to adjuvant corticosteroids, confirming that those treated tended to develop fewer kidney scars than children who were randomized to receive placebo (9.8% versus 16.8%) [12]. However, a statistically significant difference was not achieved, and the study was limited by not reaching its intended sample size.

Procalcitonin (PCT), a blood biomarker of infection, has shown good correlation to both acute pyelonephritis and kidney scarring in children [2, 13–16], with approximately 40% of children with a febrile UTI and PCT values ≥ 1 ng/mL showing kidney scars on late DMSA scan [2]. The use of PCT to identify children at high risk of kidney scarring seems a valuable strategy to select children most likely to benefit from adjuvant therapies to prevent scarring.

Based on these premises, we conducted a multicenter RCT to determine the effectiveness of adjuvant steroids in reducing kidney scar formation in young children with a first febrile UTI deemed at higher risk for kidney scarring based on their PCT values. Projections of enrollment and completion of study follow-up were calculated halfway through the study because of unanticipated low recruitment and high attrition rate. As projections showed that the intended sample size could not be reached before funding and time to complete the study ran out, an amendment to the analysis plan was approved to use Bayesian analysis. In Bayesian analyses, the probability of treatment effect (posterior probability) is estimated considering the trial data and incorporating the prior probability distribution. The prior distribution includes information on treatment effect provided by previous relevant studies (clinical trials or pilot trials), when available [17].

In this paper, we describe the original study plan and the study results using a Bayesian analysis approach.

## Methods

### Study design and participants

This multicenter RCT was conducted at five Italian hospitals. Three centers (the Ca’ Foncello Hospital of Treviso, the University Hospital of Padova, and the S. Orsola Hospital of Bologna), were part of the study since its inception, in May 2014, while two centers (the hospital of Dolo-Mirano and the University Hospital of Udine) joined the study in May 2016. The trial was approved by the Ethics Committee of the respective institutions and registered with the European Clinical Trial Database, EudraCT number: 2013-000388-10. We obtained written informed consent from parents or guardians.

Children aged 2 to 24 months with the first episode of presumed febrile UTI at high risk of kidney scarring based on PCT levels ≥ 1 ng/mL were eligible for enrolment. A presumed febrile UTI was defined as axillary temperature > 37.5°C and positive dipstick (≥ 1+ leukocyte esterase and/or nitrites) on urine samples collected by urine catheterization, in children with unexplained fever and no other signs of infection.

We excluded children who received antibiotics in 48 h before evaluation; who had known underlying kidney diseases or urinary tract abnormalities, a history of previous UTI and UTI recurrence before DMSA scan at 6 months for detection of kidney scars, a history of prematurity (birth before 36 weeks of gestational age), known immunodeficit, and contraindication to steroid therapy; or whose urine culture eventually resulted negative. Patients who were hospitalized could be approached within 48 h after starting antibiotic therapy.

### Intervention and randomization

Study participants were randomly allocated to receive dexamethasone (0.15 mg/kg per dose every 12 h for 4 days) in addition to routine care or routine care only. Dexamethasone could be administered up to 48 h after starting antibiotic therapy in case hospitalized patients could not be approached in the emergency department. Routine care consisted of oral amoxicillin-clavulanate for a total of 10 days for well-appearing children. In case of allergy or previous adverse reactions to amoxicillin-clavulanate, cefixime was administered. Routine care for ill or toxic-appearing children was intravenous ceftriaxone followed by oral antibiotic therapy after the fever had resolved for at least 48 h, for a total 10-day duration of treatment. Antibiotics could be changed according to antibiogram results on positive urine cultures or ceased if urine cultures resulted negative. The antipyretic of choice was acetaminophen at all centers, with ibuprofen recommended only if acetaminophen was ineffective in relieving the fever-related patient discomfort.

We used a computer-generated randomization list accessed through a web-based system, which was password-protected. The computerized system attributed study allocation for each consecutively enrolled patient (independently of the recruiting center). An allocation ratio of 1:1, with random block sizes of 12, was used.

### Procedures

Quantitative measurements of PCT levels were performed in blood samples drawn at the time of initial assessment.

Children randomized to the dexamethasone arm received the oral drop formulation with detailed instructions on the weight-based dose and times of administration. Patients who were discharged received a first follow-up call as soon as the result of the urine culture was available. A diagnosis of UTI was confirmed in the presence of a positive urine culture defined as the growth of only one micro-organism ≥ 50,000 CFU/mL. In children with a confirmed diagnosis of UTI, a kidney and bladder ultrasound was recommended as the standard of care.

A telephone follow-up was also conducted at 10–15 days after the diagnosis of UTI to survey the compliance with prescribed treatment and the occurrence of any possible adverse events.

At 6 months after the diagnosis of UTI, a clinical follow-up visit was performed in conjunction with a DMSA scan to detect kidney scarring. The DMSA scan was performed according to the current European guidelines of the European Association of Nuclear Medicine [18]. Kidney scarring was defined as decreased uptake with distortion of the contours or as cortical thinning with loss of parenchymal volume. Children with a positive history for UTI recurrence after the initial episode eventually identified at the 6-month follow-up were excluded from the study.

### Outcomes

The primary outcome was the presence of kidney scars on the DMSA scan performed at the 6-month follow-up. Outcome assessors were two nuclear medicine physicians, blinded to study allocation, and unaware of the patient’s clinical data, who interpreted the scans independently. Discrepancies were resolved by consensus if necessary.

Secondary outcomes were the presence of kidney scarring in the subgroup of children with higher PCT values and the acceptability of adjuvant steroid treatment in terms of the rate of discontinuation of treatment and the reported side effects.

### Statistical analysis

#### Original analysis plan

We based the original sample size calculation on the hypothesis that dexamethasone would determine a kidney scar reduction from 40% (based on the risk associated with PCT values ≥ 1 ng/mL [2]) to 20%. Sixty-four patients were required to be randomly assigned to each arm to have 80% power to detect the absolute difference of 20% in scar frequency between the groups (α = 0.05 for a one-tailed test). Estimating a 10% rate of patients who did not fulfill the criteria for UTI diagnosis (i.e., patients with negative or discordant urine cultures) and 20% of loss to follow-up, a final number of 92 patients per group were required based on the Freedman formula. We planned to summarize continuous variables as medians and interquartile ranges (IQR) and categorical data as percentages and absolute frequencies. Wilcoxon-type tests were to be used to compare continuous variables and Pearson chi-square tests, or Fisher exact tests, as appropriate, for categorical variables, considering a *p*-value of 0.05 as statistically significant.

#### Bayesian analysis plan

Projections of enrollment and completion of study follow-up showed that the intended sample size could not be reached before funding and time to complete the study ran out. For this reason, an amendment to the study protocol was approved by the participating site Ethics Committes, as well as by the Italian drugs regulatory authority (AIFA), to conduct a Bayesian analysis. The Bayesian method allows the incorporation of the available knowledge on treatment effect (translated into prior probability distribution), combining it with the trial data, such as to reduce uncertainty rather than provide a definitive response to the study hypothesis. The prior probability distribution is based on biological plausibility and on the results of other previous relevant studies, or on clinical experience [17, 19].

The sample size estimation was carried out considering a Bayesian procedure based on a Beta Binomial model for a difference in proportion outcome [20]. An average length criterion (ALC) has been considered assuming an interval coverage of 0.9 and a length of 0.35. A Beta prior has been considered for the computation based on the data from the only available RCT, at that time, on the effect of adjuvant steroids on kidney scarring [11]. Data used for the Beta prior was the proportion of kidney scarring in both the treatment and control groups. The probabilities of scarring from the RCT by Huang et al. [11] are respectively ${\overset{\hat{\mkern6mu} }{\pi}}_{treat}=0.33$ (6|18) and ${\overset{\hat{\mkern6mu} }{\pi}}_{control}=0.66$ (39|65). Based on this calculation, the informative Beta prior has been derived as *Π*_treat_ ∼ *Beta*(6, 12) and *Π*_control_ ∼ *Beta*(39, 26). The ALC achieved sample size consisted of 18 patients (9+9).

A Beta Binomial model was used to analyze the primary outcome, namely, the difference in scar proportions between the treatment and control group [21, 22]. The posterior probability distribution for the difference in proportions outcome requires the estimation of the posterior distribution of the scar proportion in each arm, separately, and was computed via resampling procedure. Given the publication of a recent very relevant study on the topic [12], we subsequently included the results of this study in the calculation of the Beta prior probability distribution, alongside the results of the trial by Huang et al. [11].

As the inference was expected to be seriously conditioned by the prior probability distribution (i.e., a priori knowledge about treatment effect incorporated in the final inference) given that only a few data points from the study were available to estimate the likelihood of treatment effect, a sensitivity analysis was performed to assess the robustness of the inferential conclusion concerning different prior distributions. In this regard, the prior probability distributions (representing the a priori knowledge on the treatment effect) considered for the analysis were as follows:

1. *Informative*, which fully incorporates in the final inference the information on the treatment effect provided by previous relevant published studies [11, 12]
2. *Low-informative*, which discounts the prior literature information weight on the final inference by 50%
3. *Uninformative*, which assumes an absolute lack of a priori knowledge on the treatment effect estimate

The final results were analyzed evaluating the alternative hypothesis that the treatment effect estimate (the difference in the proportions of kidney scar events in treatment versus control) is less than the hypothesized absolute difference of 20% in scar frequency between the groups (assuming that steroids would determine 20% fewer scars compared with the control group): margin of –0.2. The final results were also evaluated considering no difference between the groups (absolute difference of 0%): margin of 0. The analyses were conducted using R 3.6.2 [23].

Additional details on the Bayesian analysis for this study have been recently published [24] and are reported as a summary in the *Online Resource* *1**.*

## Results

### Patient characteristics

**Fig. 1:**
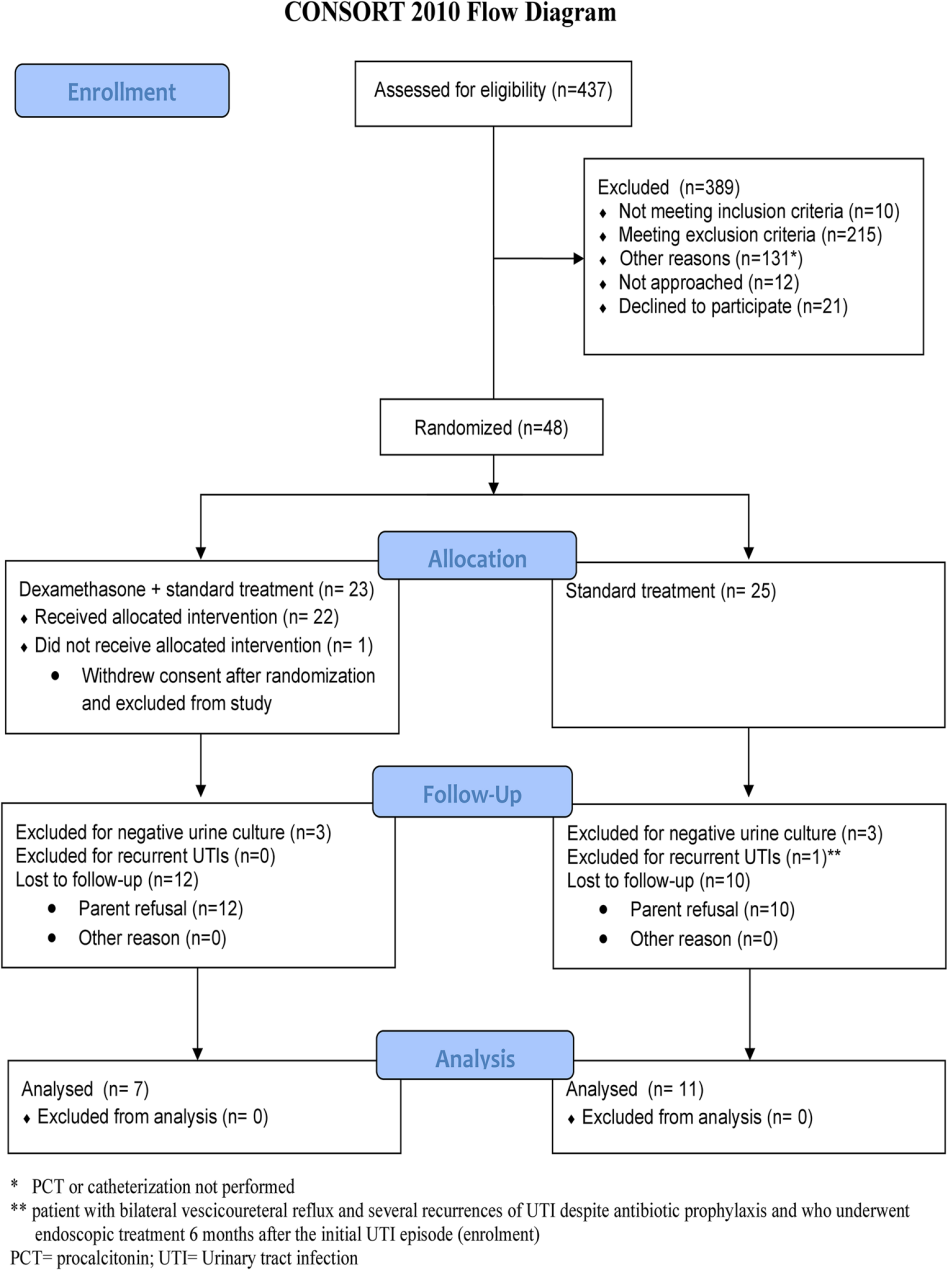
Numbers of children who were screened, allocated to the trial group, and included in the analysis

Children were recruited between May 2014 and June 2017 and follow-up completed by December 2017. A total of 437 children were assessed for eligibility, of whom 225 (51.5%) either did not meet inclusion criteria or met exclusion criteria. Of the remaining 212 patients, 131 (61.8%) did not complete the study procedures, namely, determination of serum PCT and/or urine collection through catheterization, to assess eligibility and were considered “potentially eligible.” Of the 81 eligible patients, 12 (14.8%) could not be approached by research staff and 21 (25.9%) declined consent; thus 48 (59.3%) patients underwent randomization, and 18 completed the 6-month follow-up for the primary outcome assessment and were included in the analysis (Fig. 1). The baseline demographic characteristics of children who were randomized compared with eligible and potentially eligible children were similar concerning age, sex, and race (eTable 1**-** *Online Resource* *2*). The comparison of demographic and clinical characteristics between the treatment and control group did not show significant differences (Table 1). Enrolled children were mostly younger than 1 year of age and presented after a median duration of fever of 2.5 days. Only one child had a history of urinary tract abnormality on fetal ultrasound, namely, a mild pelvic dilatation, which was not confirmed on postnatal ultrasound. Urine culture eventually yielded negative results in 6 patients (13%) who were excluded from the study, as per study protocol. The only isolated organism from positive urine cultures was *E. Coli*. Nearly 60% of patients initially received parenteral antibiotic treatment, and the overall median duration of treatment was 9.5 days. A total of 10 patients (21%) underwent a voiding cystourethrography, and vesicoureteral reflux was found in three. One of these patients had bilateral reflux and presented recurrent UTIs in the first 6 months after enrollment, which determined exclusion from the study, as per study protocol.

**Table 1:** Baseline demographic and clinical characteristics of randomized patients

Variables	Total randomized patients (*n*= 48)		*p*
	Dexamethasone group *n*= 23	Control group *n*= 25	
Age in months	9.4 (5.3–12.3)	7.4 (3.7–13.7)	0.768
Sex (females)	15 (66%)	14 (56%)	0.514
Race (Caucasian)	18 (78%)	22 (88%)	0.481
Urinary tract abnormalities on fetal US	1 (4%)	0 (0%)	0.292
Max body temperature in **°**C	39.5 (39.3–40.0)	39.3 (38.8–39.8)	0.019
Duration of fever in days	2 (2-4)	3 (2-4)	0.218
Weight in kg	8.5 (7.0–10.0)	8.0 (6.6–10.0)	0.943
Weight percentile by sex/age	50.0 (25.0–90.0)	75.0 (50.0–78.8)	0.552
Height in cm	70.0 (65.5–75.5)	68.0 (65.0–75.0)	0.752
Height percentile by sex/age	75.0 (50.0–90.0)	75.0 (50.0–90.0)	0.772
PCT ng/ml	2.8 (1.4–5.7)	3.1 (1.7–8.1)	0.677
CRP mg/L	15.0 (9.2–75)	17.0 (6.5–46.2)	0.689
Leukocytes n/mm^3^	17.920 (12.930–23.800)	18.580(14.782–24.555)	0.488
Hemoglobin g/L	11.1 (10.2–11.6)	11.0 (10.2–11.5)	0.783
Urea mg/dL	13.0 (7.3–16.8)	11.0 (8.0–16.0)	0.756
Creatinine mg/dL	0.32 (0.28–0.36)	0.30 (0.28–0.40)	0.850
Leukocyturia on urine dipstick	23 (100%)	24 (96%)	0.332
Nitraturia on urine dipstick	15 (65%)	17 (68%)	0.838
Urine method collection for culture			
•Catheterization	22 (96%)	21 (84%)	0.305
•Clean catch	1 (4%)	4 (16%)	
Hospitalization	15 (65%)	20 (80%)	0.250
Urine culture positive	20 (87%)	22 (88%)	0.772
Isolated germs on urine culture			
•*E. coli*	20 (87%)	22 (88%)	0.772
•Other germ	0 (0%)	0 (0%)	
Antibiotics initially administered			0.282
•Oral			
°Amoxicillin-clavulanate	11 (100%)	9 (100%)	
•Parenteral			
°Ceftriaxone	11 (92%)	13 (81%)	
°Amoxicillin-clavulanate	1 (8%)	1 (4%)	
°Ampicillin-sulbactam	0 (0%)	2 (8%)	
Switch in antibiotics	11 (48%)	14 (56%)	0.570
•Oral	10 (43%)	10 (40%)	
°Amoxicillin-clavulanate	6 (60%)	6 (60%)	0.410
°Cefixime	3 (30%)	2 (20%)	
°Cefpodoxime	0 (0%)	1 (10%)	
°Cefibutene	0 (0%)	1 (10%)	
°Other	1 (10%)	0 (0%)	
•Parenteral	1 (4%)	4 (16%)	
°Ampicillin-sulbactam	0 (0%)	1 (25%)	
°Ceftriaxone	1 (100%)	2 (50%)	0.660
°Meropenem	0 (0%)	1 (25%)	
Antibiotic therapy duration in days	9 (9–10)	10 (8.25–10)	0.720
Kidney and bladder US performed	18 (78%)	21 (84%)	1.000
Patients with abnormal findings	9 (50%)	4 (19%)	
°Loss of cortico-medullary differentiation	1	0	0.041
°Parenchymal thinning	0	1	
°Calyceal dilatation	4	3	
°Pelvic dilatation	4	2	
°Uretheral dilatation	2	1	
°Parenchymal hyperechogenicity	1	0	
VCUG	6 (30%)	4 (18%)	0.369
•VUR at MCUG	1 *	2 **	

*CRP* C-reactive protein, *VCUG* voiding cystourethrography, *PCT* procalcitonin, *US* ultrasound, *VUR* vesicoureteral reflux

*A patient with grade I reflux on the right side

**A patient with grade IV reflux on the left side; a patient with bilateral reflux of grade II on the right side and grade IV on the left side

The characteristics of children who completed the follow-up for the determination of the primary outcome and who were eventually included in the primary analysis are reported in Table 2. Children who were lost to follow-up were similar to those with a known outcome (**eTable 2-** *Online Resource* *2*).

**Table 2:** Baseline demographic and clinical characteristics of patients who completed the follow-up for the assessment of the primary outcome

Variables	Total randomized patients (*n*= 18)		*p*
	Dexamethasone group *n*= 7	Control group *n*= 11	
Age in months	10.7 (3.9–16.1)	11.6 (5.1–18.2)	0.964
Sex (females)	3 (43%)	7 (64%)	0.630
Race (Caucasian)	5 (71%)	9 (82%)	0.717
Urinary tract abnormalities on fetal US	1 (14%)	0 (0%)	0.389
Max body temperature in **°**C	39.5 (39.3–40.0)	39.3 (38.3–39.8)	0.099
Duration of fever in days	2 (2–4)	3 (2–4)	0.376
Weight in kg	7.7 (7.2–10.5)	8.9 (6.5–11.0)	0.856
Weight percentile by sex/age	50.0 (25.0–90.0)	75.0 (50.0–78.8)	0.824
Height in cm	68 (62.0–80.0)	70.0 (62.0–80.0)	0.892
Height percentile by sex/age	50.0 (50.0–90.0)	75.0 (50.0–90.0)	0.852
PCT ng/ml	3.6 (1.3–6.1)	2.3 (1.7–3.3)	0.526
CRP mg/L	15.2 (10.0–115.1)	17.0 (4.1–109)	0.821
Leukocytes n/mm^3^	19.630 (7.020–29.150)	18.480 (16.800–25.600)	0.441
Hemoglobin g/L	11.1 (10.2–11.7)	11.0 (10.0–11.6)	0.585
Urea mg/dL	14.0 (12.0–35.9)	11.0 (8.0–18.0)	0.266
Creatinine mg/dL	0.38 (0.33–0.50)	0.29 (0.24–0.30)	0.055
Leukocyturia on urine dipstick	6 (86%)	10 (91%)	0.732
Nitraturia on urine dipstick	6 (86%)	9 (82%)	0.829
Urine method collection for culture			
•Catheterization	7 (100%)	8 (73%)	0.130
•Clean catch	0 (0%)	3 (27%)	
Hospitalization	4 (57%)	9 (82%)	0.255
Antibiotics initially administered			0.464
•Oral			
°Amoxicillin-clavulanate	4 (57%)	3 (27%)	
•Parenteral			
°Ceftriaxone	3 (43%)	7 (64%)	
°Ampicillin-sulbactam	0 (0%)	1 (9%)	
Switch in antibiotics	3 (43%)	7 (64%)	1.000
•Oral			
°Amoxicillin-clavulanate °Cefixime	1 2	4 1	
°Cefibutene	0	1	
•Parenteral			
°Meropenem	0 (0%)	1 (9%)	
Antibiotic therapy duration in days	10 (9–10)	10 (8–15)	0.771
Kidney and bladder US performed	5 (71%)	11 (100%)	
Abnormal kidney and bladder US	4 (57%)	3 (27%)	0.627
°Parenchymal thinning	0 (0%)	1 (9%)	
°Calyceal dilatation	2 (28%)	2 (18%)	
°Pelvic dilatation	1 (14%)	2 (18%)	
°Uretheric dilatation	1 (14%)	1 (9%)	
VCUG	3 (43%)	2 (18%)	0.326
•VUR at VCUG	1 *	1 **	

*CRP* C-reactive protein, *VCUG* voiding cystourethrography, *PCT* procalcitonin, *US* ultrasound, *VUR* vesicoureteral reflux

*A patient with grade I reflux on the right side

**A patient with grade IV reflux on the left side

### Primary outcome

**Fig. 2:**
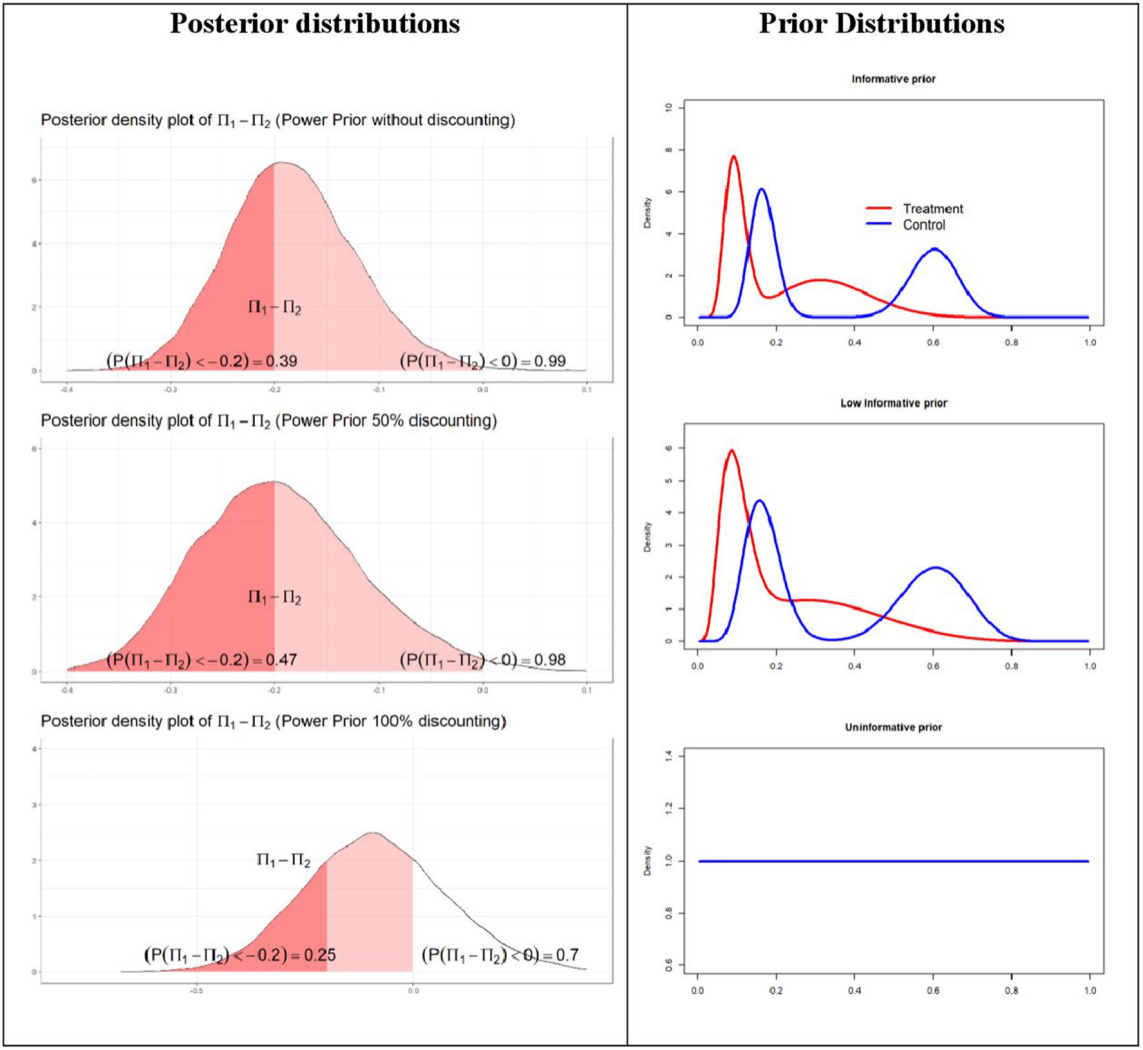
Posterior and prior distributions. The probabilities that *π*_*Treat*_ − *π*_*Control*_ are less than 0 or –0.2 are reported for informative, low-informative, and uninformative scenarios. *π*_1_ = *π*_*Treat*_ and *π*_2_ = *π*_*control*_

Of the 18 recruited patients who completed the follow-up for the study outcome, 7 were randomized to the adjuvant dexamethasone group and 11 to the control group. No kidney scars on the DMSA scan at 6 months were found in the treatment group, while two cases of kidney scarring were observed in the control arm (Table 3). Figure 2 shows the priors and the posterior probability distributions, with the probability that the difference in event rate is less than 0 (i.e., the steroid adjuvant therapy could prevent the kidney scar events) or –0.2 margin for each scenario (i.e., the steroid adjuvant therapy could reduce kidney scar formation by 20% or more). The probabilities that steroid adjuvant therapy could prevent kidney scarring (differences in proportions less than 0) are very similar and very high in the informative prior scenario (0.99) and low-informative prior (0.98), while this probability is smaller in the uninformative scenario (0.70). Considering a margin of 20% reduction, based on treatment effect, results differ across scenarios. In this case, the probabilities that steroid adjuvant therapy could reduce kidney scar formation by 20% or less are 0.61, 0.53, and 0.45 in the informative, low-informative, and uninformative setting, while the probabilities of effect beyond 20% reduction in kidney scarring are 0.39, 0.47, and 0.25, respectively, in the informative, low-informative, and uninformative setting. Considering also the 95% credibility intervals (Table 3) for the difference in proportions outcome, the interval includes the zero for the estimates calculated within the uninformative prior setting, while zero is not included for the informative and low-informative credibility interval. The –0.2 margin is instead included in all the credibility intervals (Table 3).

**Table 3:** Number and percentages of observed kidney scar events in treatment and control arm. 95% credible intervals are reported for the posterior distribution *π*_*Treat*_ − *π*_*Control*_ and for predictive posterior estimates provided in informative, low-informative, and uninformative scenarios

	Treatment (*n*=7)	Control (*n*=11)			
	0% (0)	18% (2)			
	Predictive posterior estimates (95% credible interval)		*π*_*Treat*_ − *π*_*Control*_ (95% credible interval)	*P*(*π*_*treat*_ − *π*_*control*_ < 0)	*P*(*π*_*treat*_ − *π*_*control*_ < − 0.2)
Informative	1 (0 ; 4)	4 (1 ; 7)	−0.19 (−0.29 ; −0.06)	0.99	0.39
Low-informative	0 (0 ; 3)	3 (0 ; 7)	−0.20 (−0.33 ; −0.03)	0.98	0.47
Uninformative	1 (0 ; 4)	3 (0 ; 7)	−0.09 (−0.40 ; 0.25)	0.70	0.25

The posterior predictive estimates have been also computed (Table 3). It is possible to assess that the number of posterior predictive median scar events that could have been observed if the estimated Bayesian model, which combines empirical evidence and historical information, was true is similarly very low in the treatment arm for all prior distributions. For the control group, instead, the median number of the predictive events is higher in all prior scenarios showing fewer differences between groups in the uninformative setting.

Results of the Bayesian analysis showed that a reduction in the kidney scar event rate is highly likely, when considering the effect on the final inference of the available knowledge of the adjuvant steroid therapy synthesized in the informative and low-informative prior. A preventive effect on the kidney scar event of adjuvant steroids has not been demonstrated assuming an absolute lack of a priori knowledge on the treatment effect in a full uninformative prior analysis.

### Secondary outcomes

Given the limited number of recruited patients, we were unable to assess the frequency of kidney scarring in the subgroup of children with higher PCT values. As for the acceptability of adjuvant steroid treatment, 4 out of 22 (18.2%) patients allocated to the dexamethasone group discontinued the treatment. There was only one reported side effect of transient behavioral change with reported increased irritability in the treatment arm.

## Discussion

Our study, unfortunately, failed to assess the effectiveness of adjuvant steroid treatment in reducing kidney scarring in children with acute pyelonephritis, as originally designed. Due to unanticipated significant challenges with patient recruitment and high attrition rate, we eventually used a Bayesian analysis approach to estimate the probability of the treatment effect. Bayesian analysis has been previously used for trials where difficult recruitment was expected [25]. The Bayesian analysis in our study showed a 99% probability of any reduction in kidney scarring in children treated with adjuvant steroids using an informative prior probability distribution based on the results of the studies by Huang et al. [11] and by Shaikh et al. [12] and 98% using a low-informative prior. The probability of steroid effect decreased to 70% when using an uninformative prior probability distribution, which assumes an absolute lack of a priori knowledge on the treatment effect. However, this assumption, which is the most conservative within the sensitivity analysis, is the least realistic, as it does not consider the available published evidence that our results build upon. The probability that steroid adjuvant therapy could reduce kidney scar formation by 20% or less was higher than 50% in the informative and low-informative scenarios (61% and 53%, respectively), while it decreased to 45% in the uninformative scenario. Overall, our results go in the same direction as the two previously published studies on the topic [11, 12].

The Taiwanese RCT by Huang et al. [11] enrolled a small sample of children within a wide age range and a high risk of kidney scarring based on radiologically confirmed extensive pyelonephritis on acute DMSA scan, with an unbalanced ratio between the study arms (1:3.4 of treatment versus control group). These factors likely explain the nearly 50% reduction in scar development found by this study. The North American RCT by Shaikh et al. [12], although the largest to date with 254 patients with complete follow-up, failed to reach the intended sample size of 320 children to detect a 10% absolute reduction in kidney scarring. The study, which was completed over 7 years, presents some important differences compared with ours. It included children within a broader age range (between 2 months and 6 years), it did not select patients on the basis of PCT values, it did not exclude children with recurrent UTIs, and it allowed for a broader window for the assessment of the primary outcome (DMSA scan between 5 and 24 months) and planned a shorter course of dexamethasone (3 days instead of 4) at the same daily dosage as in our study. This study, which included a much larger sample than ours, encountered some similar challenges. Approximately 50% of eligible patients declined participation, nearly 30% were excluded post-randomization for a negative urine culture result, and one-third of retained patients failed to complete follow-up for the assessment of the primary outcome.

Successful recruitment and retention of patients in clinical trials are known to be some of the greatest challenges in conducting RCTs. In our trial, we similarly found many barriers to both recruitment and retention. First, adherence to study procedures by clinicians was a significant challenge to patient recruitment in our study. Failure to perform blood tests for PCT determination prevented the assessment of eligibility for the study, with PCT ≥ 1 ng/mL being one of the inclusion criteria. Clinicians often felt that determination of blood tests, although part of local management protocols, was time-consuming and was of limited added value in well-appearing previously healthy children with a first uncomplicated UTI episode. The role and yield of blood markers in the management of first febrile UTIs episodes are debatable and are not routinely recommended by the Italian guidelines, the National Institute of Care Excellence (NICE), and the American Academy of Pediatrics (AAP) guidelines [3, 26–29]. While there is general agreement that blood tests should be performed in infants younger than 2 or 3 months, our study included only children older than 2 months. Given the association of PCT values with kidney scarring, the inclusion of patients based on PCT values was considered by experts, at the time of study design, the best strategy to select patients most likely to benefit from adjuvant steroid treatment [2, 30]. A subsequent meta-analysis of individual patient data showed that children with an abnormal kidney ultrasound or with a combination of high fever (≥ 39°C) and an etiologic organism other than *E. Coli* are at high risk for the development of kidney scarring [31]. However, all these data are not available at the time of initial assessment, when the administration of steroids should be started to maximize their effect during the acute inflammatory phase. Second, we experienced a high percentage of declined consent to study participation due to parental concerns and fear of administering steroids to their children. This was unanticipated, given the widespread use and acceptance of steroid administration in asthma and croup in the acute care setting. However, the 4-day course with a twice a day administration schedule of dexamethasone may have induced parents to think this was a high dose treatment and could have discouraged participation for fear of side effects. A structured qualitative analysis of parental views and concerns would have helped to gain an accurate insight into factors preventing participation in the study. Third, adherence of participants to study procedures was another main obstacle to completion of follow-up, which was achieved by only 45% of enrolled subjects. Parents were reluctant for their children to undergo the DMSA scan at 6 months, as they had been well from the initial UTI episode and the exam was perceived as invasive for their children. In addition, many parents reported they were discouraged from having their children undergo the scan, after talking with other physicians. Based on the Italian guidelines for the management of UTI, published just after our study protocol was funded and approved [26, 32], a DMSA scan is recommended in the presence of pre-defined abnormalities on ultrasound, clinical risk factors for more severe infections, or in the case of a second febrile UTI. PCT values were not mentioned in the guidelines as a risk factor for more severe infections. While our team completed prior studies, including a large RCT, on the effectiveness of oral versus parenteral antibiotics for acute pyelonephritis and diagnostic accuracy of PCT for acute pyelonephritis and kidney scars [1, 13] DMSA scan at that time was part of the routine management of UTIs both acutely and at 6 months, and no additional treatment was under investigation other than antibiotic therapy. Based on the above considerations, it appears very unlikely that another RCT on the effect of adjuvant steroids on kidney scarring in children could be successfully carried out in Italy in the future. Similarly, based on the results of the recent North American study, future RCTs with the same objective are unlikely to be successfully completed in a reasonable time frame also in settings with higher resources available for clinical research. However, given the important potential implications of a cheap and easily implementable therapy, such as steroids, in improving the health outcomes of children with UTI, with a potential greater impact in those children with underlying kidney diseases or urinary tract abnormalities, probabilities of effect may be helpful to guide clinical practice. With this respect, the Bayesian approach is able to provide clinicians with probabilities that the clinical effect lies in a particular range and can be thus used in decision-making. As reported by Lilford et al. [19], “the strength of the Bayesian approach is that it produces a probability distribution which may guide clinical action even when a "definitive" answer is not available … Clinicians are familiar with the need to make decisions under uncertainty and recommend the treatment which seems to have the best chance of maximizing benefit (expected utility) … Nevertheless, a decision taken on the basis of a posterior belief that includes evidence from a randomized controlled trial, however small, is more likely to be correct than a decision based simply on a prior belief with no evidence from such a trial. Any randomized evidence is better than none.”

As reported above, our study suffered from many limitations that prevented its completion as per original design. In addition, some patients may have received one or more doses of ibuprofen for the treatment of their fever, which might have had an influence on the study outcome. However, the study steering committee made a pragmatic choice not to deviate from the standard of care on fever management at participating sites to avoid parental confusion with respect to antipyretic administration for possible future febrile illnesses. In making this decision, the study steering committee considered that randomization would equally distribute known and unknown confounding factors between the study arms. Furthermore, the study sample size was achieved with respect to the Bayesian design; however, the allocation in the two treatment groups appears to be unbalanced (11 versus 7). Despite all these limitations, our Bayesian analysis could provide probabilities of treatment effect that could be used in clinical practice.

## Conclusions

Conducting a trial to assess the effectiveness of adjuvant steroid treatment in reducing kidney scar development in children with acute pyelonephritis has proven challenging using a frequentistic approach. A Bayesian analysis approach showed that adjuvant steroids are very likely to reduce kidney scarring, with a more than 50% probability to reduce kidney scar formation by up to 20%, in the setting of an informative or low-informative prior probability distribution.

## Supplementary Information

ESM 1 (PDF 187 kb)

ESM 2 (PDF 237 kb)

ESM 3 (PPTX 44 kb)

## Data Availability

The datasets generated during and/or analyzed during the current study are available from the corresponding author on reasonable request.
