# Supplementary material for: Oral steroids for reducing kidney scarring in young children with febrile urinary tract infections: the contribution of Bayesian analysis to a randomized trial not reaching its intended sample size
Source: Pediatr Nephrol. 2021 May 25;36(11):3681–92. doi: 10.1007/s00467-021-05117-5 (PMC8497283; doi:10.1007/s00467-021-05117-5)
Supplement: Supplementary file 1 — (PDF 187 kb) [file 467_2021_5117_MOESM1_ESM.pdf]

Supplementary information- *Online Resource 1*

Title:

Oral steroids for reducing renal scarring in young children with febrile urinary tract infections: the contribution of Bayesian analysis to a randomized trial not reaching its intended sample size

Authors:

Liviana Da Dalt[a*], Silvia Bressan[a*], Floriana Scozzola[b], Enrico Vidal[a,c], Monia Gennari[d], Claudio La Scola[e], Mauro Anselmi[f], Elisabetta Miorin[c], Pietro Zucchetta[g], Danila Azzolina[h], Dario Gregori[h], Giovanni Montini[i,l]

* Joint first authors

Affiliations:

[a]Department of Women's and Children's Health, University of Padova, Padova, Italy;

[b] Pediatric Unit, Treviso Hospital, Treviso, Italy;

[c] Division of Pediatrics, Department of Medicine (DAME), University Hospital of Udine, Italy;

[d] Pediatric Emergency Unit, Department of Medical and Surgical Sciences (DIMEC), S. Orsola Hospital, Bologna, Italy;

[e] Nephrology and Dialysis Unit, Department of Woman, Child and Urological Diseases, Azienda Ospedaliero-Universitaria Sant'Orsola-Malpighi, Bologna, Italy

[f]Pediatric Unit, Dolo-Mirano Hospital, Dolo, Italy;

[g] Nuclear Medicine Unit, Department of Medicine DIMED, University-Hospital of Padova, Padova, Italy

[h] Unit of Biostatistics, Epidemiology and Public Health, Department of Cardiac, Thoracic, Vascular Sciences and Public Health, University of Padova, Padova, Italy;

[i] Pediatric Nephrology, Dialysis and Transplant Unit, Fondazione IRCCS Ca' Granda, Ospedale Maggiore Policlinico, Milano, Italy;

[l] Giuliana and Bernardo Caprotti Chair of Pediatrics, Department of Clinical Sciences and Community Health, University of Milano, Milano, Italy.

Corresponding author:

Silvia Bressan

Department of Women's and Children's Health, University of Padova

Via Giustiniani 4, 35128, Padova (Italy)

silvia.bressan.1@unipd.it; silviabress@gmail.com

Tel: +39 0498213570.

Technical appendix

A Bayesian analysis of the renal scar event rate was prespecified to estimate the probability of treatment benefit considering the recommendations for trials conducted with a limited sample size in frequentist design (1). A Beta-binomial model was used to analyze the difference in scar proportions between arms (2).

A Mixture of Beta priors has been considered for the outcome evaluation using the data provided by literature (3,4). Two clinical trial results have been combined in a mixture of distributions.

1. Huang et al. (3) the study reports a probability of scarring of $\hat{\pi}_{treat\ (Huang)} = 0.33$ (6|18) and $\hat{\pi}_{control\ (Huang)} = 0.66$ (39|65) respectively in treatment and control arm. Considering this information, informative Beta prior has been derived as:

$$\Pi_{\text{treat (Huang)}} \sim Beta(6,12)$$

$$\Pi_{\text{control (Huang)}} \sim Beta(39,26)$$

2. Shaikh et al. (4) study reports, instead, a probability of scarring of $\hat{\pi}_{treat\ (Shaikh)} = 0.098$ (12|123) and $\hat{\pi}_{control\ (Shaikh)} = 0.168$ (22|131) respectively in treatment and control arm. Considering this information, informative Beta prior has been derived as:

$$\Pi_{\text{treat (Shaikh)}} \sim Beta(12,111)$$

$$\Pi_{\text{control(Shaikh)}} \sim Beta(22,109)$$

The information has been combined in a mixture of Beta prior:

$$\Pi_{\text{treat}} = p\Pi_{\text{treat (Huang)}} + (1-p)\Pi_{\text{treat (Huang)}}$$

$$\Pi_{\text{control}} = p\Pi_{\text{control (Huang)}} + (1-p)\Pi_{\text{control (Huang)}}$$

An equal weight ($p = 0.5$) to the components of the mixture prior has been assumed.

The posterior distribution for the difference in proportions outcome requires the estimation of the posterior distribution of the scar proportion in each arm, separately, and has been computed with the following resampling procedure(5): A first resampling of the proportion of scarring π^*_{treat} from $\pi_{treat}|X_{treat}$ which is the posterior distribution for the treatment group;

1. A second resampling of $\pi^*_{control}$ from $\pi_{control}|X_2$;
2. A posterior distribution, for the parameter related to the difference in proportions, has been obtained by calculating $\pi^*_{treat} - \pi^*_{control}$ from the previously resampled distributions(6).

Resampling procedures were performed using an MCMC estimation algorithm, as indicated in the literature (5), using 3 chains, 5000 iterations, and 1000 adaptations. Computations were performed using OpenBUGS (7) and R version 3.3.2 (8).

Sensitivity Analysis

The inference was expected to be seriously conditioned by the prior choice, as only a few data points were available to estimate the likelihood. For this reason, a sensitivity analysis was performed to assess the robustness of the inferential conclusion concerning the different prior choices.

Different levels of penalization (discounting) may be provided for the historical information using a power prior approach (9) to perform a sensitivity analysis on the prior choices. The historical information may be included in the final inference using a $Beta(\alpha_1, \beta_1)$ prior, where:

$$\alpha_1 = \alpha_0 d_0 + 1$$

$$\beta_1 = \beta_0 d_0 + 1$$

The α_0 and $\beta_0$values are the parameters defined by the number of successes and failures derived from the literature and are $(\alpha_0 - 1)$ and $(\beta_0 - 1)$, respectively. The value d_0 defines the amount of historical information to be included in the final inference. The discounting factor is otherwise defined as $(1 - d_0) \times 100$ and represents the levels of penalization (discounting) on the historical information derived from other studies.

1. If d_0 = 0, the data provided by the literature are not considered, indicating a 100% discount on the historical information. According to this scenario, the prior is an uninformative $Beta(1,1)$ distribution.
2. If d_0=1, all the information provided by the literature is considered in the inference, indicating a 0% discount on the historical data.

In this setting, three different scenarios were hypothesized for the prior computation:

- **Power Prior without discounting (Informative,** d_0=1**).** A Beta informative prior was derived considering the number of successes and failures found in the literature (10), defining prior probability distributions as a $Beta(6, 12)$ and a $Beta(39,26)$ for the first component of the mixture of priors (Huang et. al. prior), and a $Beta(12,111)$ and a $Beta(22,109)$ for the second element of the mixture.
- **Power Prior 50% discounting (Low-Informative,** d_0=0.5**).** The Beta prior with a 50% discount, defined in the literature as a Substantial-Moderate discounting factor (11), was defined on the Beta parameters composing the mixture of priors specified in the informative scenario.
- **Power Prior 100% discounting (Uninformative,** d_0=0**).** Defines a mixture of $Beta(1,1)$ prior.

References

1. Lilford RJ, Thornton J, Braunholtz D (1995). Clinical trials and rare diseases: a way out of a conundrum. BMJ 311:1621–5.

2. Albert J (2009). Bayesian computation with R. Springer Science & Business Media.

3. Huang YC, Lin YC, Wei CF, Deng WL, Huang HC (2016). The pathogenicity factor HrpF interacts with HrpA and HrpG to modulate type III secretion system (T3SS) function and t3ss expression in Pseudomonas syringae pv. averrhoi. Mol Plant Pathol 17:1080–94.

4. Shaikh N, Shope TR, Hoberman A, et al (2020). Corticosteroids to prevent kidney scarring in children with a febrile urinary tract infection: a randomized trial. Pediatr Nephrol 35:2113-2120

5. Kawasaki Y, Shimokawa A, Miyaoka E (2013). Comparison of three calculation methods for a Bayesian inference of P (π1> π2). J Mod Appl Stat Methods 12:15.

6. Barry J (2011). Doing Bayesian data analysis: A tutorial with R and BUGS. Eur J Psychol 7:778.

7. Lunn D, Spiegelhalter D, Thomas A, Best N (2009). The BUGS project: Evolution, critique and future directions. Stat Med 28:3049–67.

8. R Core Team. R: A Language and Environment for Statistical Computing [Internet]. Vienna, Austria: R Foundation for Statistical Computing; 2015. Available at: https://www.R-project.org/

9. Ibrahim JG, Chen M-H (2000). Power prior distributions for regression models. Stat Sci 15:46–60.

10. Huang YY, Chen MJ, Chiu NT, et al (2011). Adjunctive Oral Methylprednisolone in Pediatric Acute Pyelonephritis Alleviates Renal Scarring. Pediatrics 128:e496-504

11. Santis FD (2007). Using historical data for Bayesian sample size determination. J R Stat Soc Ser A Stat Soc 170:95–113.
