# Supplementary material for: Oral steroids for reducing kidney scarring in young children with febrile urinary tract infections: the contribution of Bayesian analysis to a randomized trial not reaching its intended sample size
Source: Pediatr Nephrol. 2021 May 25;36(11):3681–92. doi: 10.1007/s00467-021-05117-5 (PMC8497283; doi:10.1007/s00467-021-05117-5)
Supplement: Supplementary file 2 — (PDF 237 kb) [file 467_2021_5117_MOESM2_ESM.pdf]

Supplementary Tables- *Online Resource 2*

Title:
Oral steroids for reducing renal scarring in young children with febrile urinary tract infections: the contribution of Bayesian analysis to a randomized trial not reaching its intended sample size

Authors:
Liviana Da Dalt[a*], Silvia Bressan[a*], Floriana Scozzola[b], Enrico Vidal[a,c], Monia Gennari[d], Claudio La Scola[e], Mauro Anselmi[f], Elisabetta Miorin[c], Pietro Zucchetta[g], Danila Azzolina[h], Dario Gregori[h], Giovanni Montini[i,l]

* Joint first authors

Affiliations:
[a]Department of Women's and Children's Health, University of Padova, Padova, Italy;
[b] Pediatric Unit, Treviso Hospital, Treviso, Italy;
[c] Division of Pediatrics, Department of Medicine (DAME), University Hospital of Udine, Italy;
[d] Pediatric Emergency Unit, Department of Medical and Surgical Sciences (DIMEC), S. Orsola Hospital, Bologna, Italy;
[e] Nephrology and Dialysis Unit, Department of Woman, Child and Urological Diseases, Azienda Ospedaliero-Universitaria Sant'Orsola-Malpighi, Bologna, Italy
[f]Pediatric Unit, Dolo-Mirano Hospital, Dolo, Italy;
[g] Nuclear Medicine Unit, Department of Medicine DIMED, University-Hospital of Padova, Padova, Italy
[h] Unit of Biostatistics, Epidemiology and Public Health, Department of Cardiac, Thoracic, Vascular Sciences and Public Health, University of Padova, Padova, Italy;
[i] Pediatric Nephrology, Dialysis and Transplant Unit, Fondazione IRCCS Ca' Granda, Ospedale Maggiore Policlinico, Milano, Italy;
[l] Giuliana and Bernardo Caprotti Chair of Pediatrics, Department of Clinical Sciences and Community Health, University of Milano, Milano, Italy.

Corresponding author:
Silvia Bressan
Department of Women's and Children's Health, University of Padova
Via Giustiniani 4, 35128, Padova (Italy)
silvia.bressan.1@unipd.it; silviabress@gmail.com
Tel: +39 0498213570.

eTable 1. Comparison of baseline demographic characteristics between enrolled patients and eligible plus potentially eligible patients.

	**n = 48**	**n = 164***	**p**
**Age in months - median, (IQR)**	8.3 (4.2-12.9)	9.1 (6.1-13.4)	0.259
**Sex (females) – n (%)**	29 (60%)	108 (70%)	0.231
**Race (caucasian) – n (%)**	40 (83%)	130 (84%)	0.924

* sum of potentially eligible children (n=131, who did not undergo PCT determination or urine catheterization for urine specimen collection) and eligible children (declined consent, n=21; and not approached, n=12)

eTable 2. Baseline demographic and clinical characteristics of patients who completed the follow up for the assessment of the primary outcome and those who were lost to follow up

Variables	**Total randomized patients (n= 48)<br>Patients excluded after randomization (n=8§)**		p
	**Included in the final analysis<br>n= 18**	**Lost to follow up<br>n= 22**	
Age in months	7.2 (3.9-15.9)	9.1 (5.3-12.6)	0.891
Sex (females)	10 (56%)	14 (64%)	0.604
Race (caucasian)	15 (83%)	20 (91%)	0.515
Urinary tract abnormalities on fetal US	0 (0%)	0 (0%)	na
Max body temperature in °C	39.4 (39.0-40.0)	39.7 (39.3-40.0)	0.179
Duration of fever in days	2.5 (2-4)	2.5 (2-4)	0.944
Weight in kg	8.3 (6.6-10.9)	9.0 (7.0-10.0)	0.935
Weight percentile by sex/age	50.0 (25.0-90.0)	75.0 (50.0-95.0)	0.176
Height in cm	69.0 (62.0-80.0)	70.5 (65.0-76.0)	0.860
Height percentile by sex/age	75.0 (50.0 -90.0)	75.0 (50.0 -90.0)	0.338
PCT ng/ml	2.6 (1.7-6.1)	2.8 (1.3 – 8.1)	0.891
CRP mg/L	16.75 (10.0-109)	10.2 (6.9-70.0)	0.464
Leukocytes n/mm^3	18.100 (8.680-23.650)	16.740(12.510-23.000)	0.927
Hemoglobin g/L	11.1 (10.1-11.6)	11.2 (10.7-11.7)	0.532
Urea mg/dL	13.0 (9.1-16.5)	10.6 (7.0-16.0)	0.519
Creatinine mg/dL	0.32 (0.28-0.39)	0.31 (0.26-0.36)	0.470
Leukocyturia on urine dipstick	18 (100%)	22 (100%)	na
Nitraturia on urine dipstick	15 (83%)	15 (68%)	0.271
Hospitalization	13 (72%)	15 (68%)	0.701
Antibiotics initially administered			0.781
• Oral			
o Amoxicillin –clavulanate	7 (39%)	10 (46%)	
• Parenteral			
o Ceftriaxone	10 (56%)	10 (46%)	
o Amoxicillin –clavulanate	0 (0%)	1 (5%)	
o Ampicillin - sulbactam	1 (6%)	1 (5%)	
Antibiotic therapy duration in days	10 (10-10)	10 (9-10)	0.085
Renal and bladder US performed	16 (89%)	20 (91%)	1.000
Patients with abnormal findings	7 (44%)	5 (25%)	
o Loss of cortico-medullary differentiation	0	1	
o Parenchymal thinning	1	0	
o Parenchymal hyperechogenicity	0	1	
o Calyceal dilatation	4	3	
o Pelvic dilatation	3	3	
o Uretheral dilatation	2	1	

§ 6 patients eventually had a negative urine culture, one patient withdrew consent after randomization and one had recurrent UTIs.
