# Supplementary material for: Oral steroids for reducing kidney scarring in young children with febrile urinary tract infections: the contribution of Bayesian analysis to a randomized trial not reaching its intended sample size
Source: Pediatr Nephrol. 2021 May 25;36(11):3681–92. doi: 10.1007/s00467-021-05117-5 (PMC8497283; doi:10.1007/s00467-021-05117-5)
Supplement: Supplementary file 3 — (PPTX 44 kb) [file 467_2021_5117_MOESM3_ESM.pptx]

## Slide 1
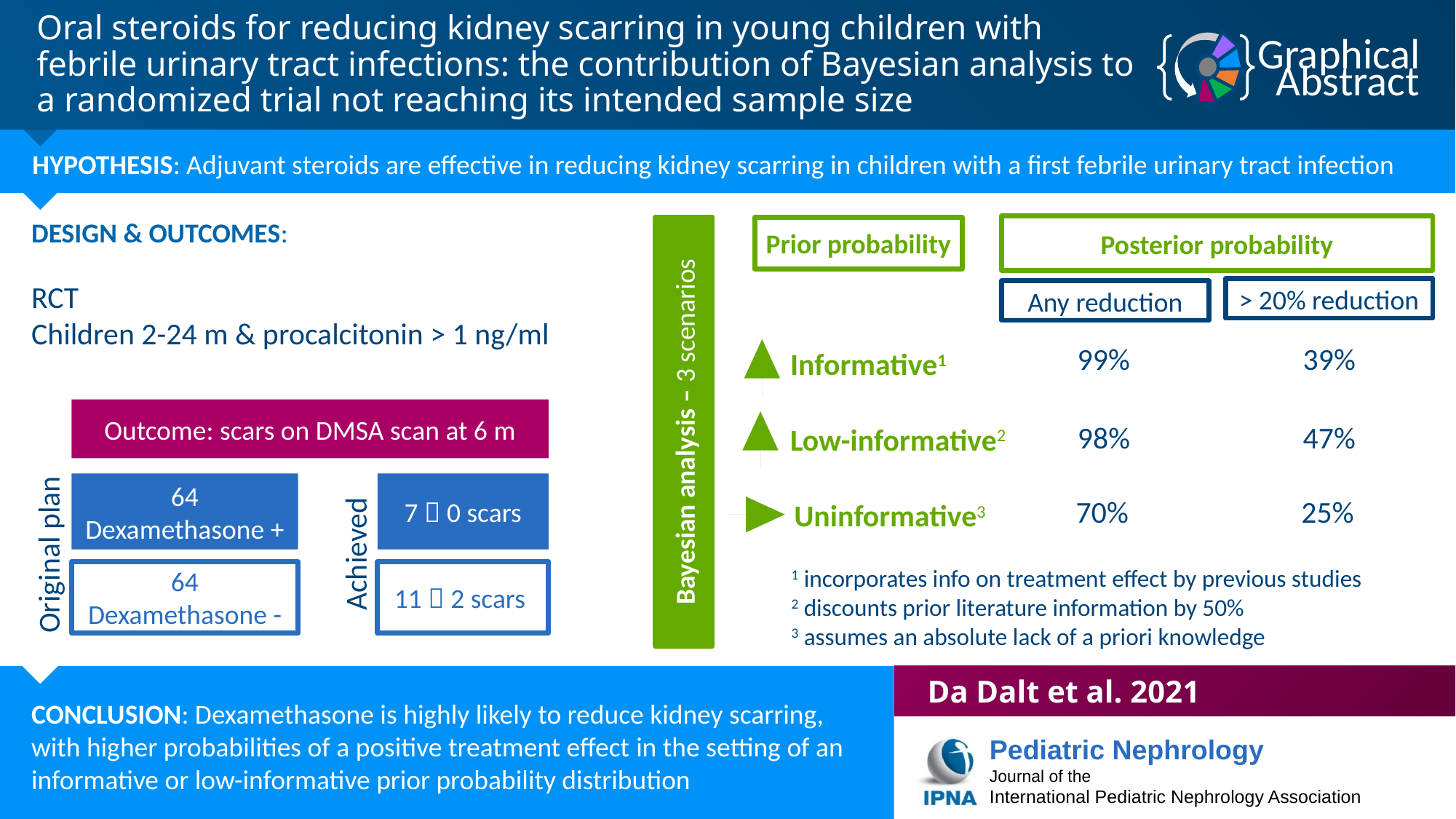

Oral steroids for reducing kidney scarring in young children with febrile urinary tract infections: the contribution of Bayesian analysis to a randomized trial not reaching its intended sample size
HYPOTHESIS: Adjuvant steroids are effective in reducing kidney scarring in children with a first febrile urinary tract infection
DESIGN & OUTCOMES:
Posterior probability
Prior probability
RCT
Children 2-24 m & procalcitonin > 1 ng/ml
> 20% reduction
Any reduction
39%
99%
Informative1
Outcome: scars on DMSA scan at 6 m
Bayesian analysis – 3 scenarios
47%
98%
Low-informative2
64 Dexamethasone +
7  0 scars
25%
70%
Uninformative3
Original plan
Achieved
1 incorporates info on treatment effect by previous studies
2 discounts prior literature information by 50%
3 assumes an absolute lack of a priori knowledge
64
Dexamethasone -
11  2 scars
Da Dalt et al. 2021
CONCLUSION: Dexamethasone is highly likely to reduce kidney scarring, with higher probabilities of a positive treatment effect in the setting of an informative or low-informative prior probability distribution
